# Relationship of apolipoprotein(a) isoform size with clearance and production of lipoprotein(a) in a diverse cohort

**DOI:** 10.1016/j.jlr.2023.100336

**Published:** 2023-01-24

**Authors:** Anastasiya Matveyenko, Nelsa Matienzo, Henry Ginsberg, Renu Nandakumar, Heather Seid, Rajasekhar Ramakrishnan, Steve Holleran, Tiffany Thomas, Gissette Reyes-Soffer

**Affiliations:** 1 Department of Medicine, Columbia University Vagelos College of Physicians and Surgeons, New York, NY, USA; 2 Irving Institute for Clinical and Translational Research, Columbia University, New York, NY, USA; 3 Center for Biomathematics, Department of Pediatrics, Columbia University Vagelos College of Physicians and Surgeons, New York, NY, USA; 4 Department of Pathology and Cell Biology, Columbia University Vagelos College of Physicians and Surgeons, New York, NY, USA

**Keywords:** kringle IV Type 2 repeats (KIV-2), Weighted isoform size (*wIS*), Apolipoprotein (a) production, Apolipoprotein (a) fractional clearance, Stable isotope studies, Atherosclerotic cardiovascular disease, Self-reported race and ethnicity (SRRE), Lipoprotein(a) concentration, Lipoprotein(a) circulation, ASCVD, atherosclerotic cardiovascular disease, ASO, antisense oligonucleotide, FCR, fractional clearance rate, KIV-2, kringle IV type 2, Lp(a), lipoprotein(a), PR, production rate, SRRE, self-reported race and ethnicity, TG, triglyceride, *wIS*, weighted isoform size

## Abstract

Lipoprotein(a) [Lp(a)] has two main proteins, apoB100 and apo(a). High levels of Lp(a) confer an increased risk for atherosclerotic cardiovascular disease. Most people have two circulating isoforms of apo(a) differing in their molecular mass, determined by the number of Kringle IV Type 2 repeats. Previous studies report a strong inverse relationship between Lp(a) levels and apo(a) isoform sizes. The roles of Lp(a) production and fractional clearance and how ancestry affects this relationship remain incompletely defined. We therefore examined the relationships of apo(a) size with Lp(a) levels and both apo(a) fractional clearance rates (FCR) and production rates (PR) in 32 individuals not on lipid-lowering treatment. We determined plasma Lp(a) levels and apo(a) isoform sizes and used the relative expression of the two isoforms to calculate a “weighted isoform size” (*wIS*). Stable isotope studies were performed, using D3-leucine, to determine the apo(a) FCR and PR. As expected, plasma Lp(a) concentrations were inversely correlated with *wIS* (*R*^2^ = 0.27; *P* = 0.002). The *wIS* had a modest positive correlation with apo(a) FCR (*R*^2^ = 0.10, *P* = 0.08) and a negative correlation with apo(a) PR (*R*^2^ = 0.11; *P* = 0.06). The relationship between *wIS* and PR became significant when we controlled for self-reported race and ethnicity (SRRE) (*R*^2^ = 0.24, *P* = 0.03); controlling for SRRE did not affect the relationship between *wIS* and FCR. Apo(a) wIS plays a role in both FCR and PR; however, adjusting for SRRE strengthens the correlation between wIS and PR, suggesting an effect of ancestry.

Lipoprotein(a) [Lp(a)] has two main protein components: each particle has one apolipoprotein B100 (apoB100) molecule covalently bound to one apo(a) molecule (1, 2). High levels of Lp(a) are causal for atherosclerotic cardiovascular disease (ASCVD) (3), as confirmed by large epidemiological studies (4), genome-wide association studies (5, 6), and Mendelian randomization studies (7, 8) .

A distinct feature of this apoB100-containing lipoprotein is the variability of apo(a) size, with masses that range from 300 to 800 kDa (9, 10), due to the number of Kringle IV type 2 (KIV-2) repeats ranging from 1 to > 40 (11, 12). Most people express two different apo(a) isoforms and these are synthesized in the liver.

Previous studies have shown a consistent and strong inverse relationship between plasma Lp(a) concentration and isoform size (13, 14); high Lp(a) levels are associated with low numbers of KIV-2 repeats and small isoforms, whereas low Lp(a) levels are associated with high numbers of KIV-2 repeats and large isoforms. The KIV-2 repeat size polymorphism explains approximately 30%–70% of the variance in Lp(a) levels (15). What regulates this relationship is not clear. The levels of Lp(a) in plasma are determined by the rate of entry of these particles into the circulation (production rate: PR) and the efficiency of their removal (fractional clearance rate: FCR). Several studies have examined the association of Lp(a) concentration with FCR and PR, with data suggesting that either or both play a role in the regulation of Lp(a) levels in the circulation (16, 17, 18, 19, 20, 21). Some of these studies also interrogated the associations of circulating levels of individual apo(a) isoform sizes with FCR and PR. In two of the studies, isoform size was tightly associated with PR: smaller isoforms with fewer KIV-2 repeats have higher PRs (18, 20). However, two other studies showed that both FCR and PR were affected by isoform size (19, 21). Most of these studies were conducted in predominantly Caucasian populations and have not taken self-reported race and ethnicity (SRRE) differences into account. It is well established that Lp(a) concentrations differs by SRRE (22, 23). To gain additional insights into this issue, we combined data from our previously completed studies of the effects of pharmacologic interventions on the kinetics of apo(a), considering the SRRE of the subjects in those studies (24, 25, 26). Our results, using a weighted isoform size (*wIS*) for each subject, support previous studies indicating the isoform size is more closely related to PR than to FCR and that ancestry, as assessed by SRRE, impacts the association between isoform size and PR.

## MATERIALS and METHODS

### Study population

The study subjects had participated in one of three separate stable isotope studies examining the effects on lipoprotein metabolism of (1) an apoB100 antisense, (2) an inhibitor of cholesteryl ester transfer protein, or (3) a monoclonal antibody against PCSK9 (24, 25, 26). The studies were approved by the Columbia University Irving Medical Center Institutional Review Board. All subjects provided informed consent before enrolling in the studies, which included consent for the use of their study data and samples for future research. Due to mass spectrometry assay sensitivity limitations, we only included subjects with Lp(a) concentrations above 10 nmol/L. The present analysis uses the baseline (preintervention phase) studies of 32 healthy individuals of varying SRRE. Subjects were neither on lipid-lowering agents, nor were they taking over-the-counter supplements. None of the subjects had clinical ASCVD and were considered in good health as assessed by medical history and physical exam. The studies reported in this manuscript abide by the Declaration of Helsinki.

### Study design

Complete details of the stable isotope studies on the metabolism of apoB100 and apo(a) have been previously published (24, 25, 26). Briefly, isocaloric, low-fat liquid meals (57% carbohydrate, 18% fat, and 25% protein) were started 8 hours (h) before stable isotope administration (1:00 AM on Day 1) and provided to subjects every 2 h for the next 32 h to maintain steady state metabolic conditions during the kinetic studies. Subjects received a bolus injection of 5,5,5-D_3_-leucine dissolved in 0.15 M NaCl (10 μmol/kg body weight) immediately followed by a constant infusion of D_3_-leucine dissolved in 0.15 M NaCl (10 μmol/kg body weight/hour) for 15 h. EDTA blood samples were collected at 18 predefined times over 24 h and plasma separated and stored at −80°C. Aliquots of these banked samples were utilized for this study; the samples had not been previously thawed or refrozen.

### Biochemical and immunological assays

Plasma lipids [total cholesterol (C), triglycerides (TGs), and high density lipoprotein (HDL)-C] were measured on an Integra400plus (Roche) from samples obtained at baseline. Plasma low density lipoprotein (LDL)-C levels were calculated using the Friedewald formula (no subject had a TG level >400 mg/dl). Plasma apoB100 levels were measured by a ELISA kit # 3715-1HP-2, from Mabtech, Inc, Cincinnati, OH.

### Apo(a) stable isotope enrichment determination

Apo(a) enrichment with D_3_-leucine was measured as described by Zhou *et al.* (27). In brief, 200 μl of the LDL fraction or equal volumes of LDL (100 μl) and HDL (100 μl) fractions isolated from plasma by ultracentrifugation were desalted. Isolated lipoprotein fractions were then treated with dithiothreitol to open disulfide bonds, alkylated with iodoacetamide, and digested using trypsin. A multiple reaction monitoring method was used to monitor the following precursor-product ion transitions of a peptide specific to apo(a): (LFLEPTQADIALLK): 786.7 > 1069.7 (M0) and 788.2 > 1069.7 (M3). Two microliteres of the digested samples were analyzed using a nanoAcquity ultra-performance LC system coupled with an ionKey source integrated to a Xevo TQ-S triple quadrupole tandem mass spectrometer (Waters, Milford, Massachusetts). The separation was achieved using an iKey Peptide BEH C18 separation device (130 Ǻ, 1.7 μm, 150 μm × 100 mm) maintained at 60°C. The gradient was 90% A (0.1% formic acid in water)/10% B (0.1% formic acid in acetonitrile) ramped linearly to 10% A at 6 min, held for 3 min, and then reequilibrated to initial conditions (total run time: 12 min; flow rate: 3 μl/min). The multiple reaction transitions were monitored with a collision energy of 24 eV.

### Lp(a) concentration and apo(a) isoform size

Lp(a) plasma concentration was measured using the isoform-independent sandwich ELISA developed by the Northwest Lipid Metabolism and Diabetes Research Laboratory (28). Apo(a) isoform size measurements were performed by the same laboratory. We started with 250 μl of plasma and each sample was diluted in saline to have 100 ng of protein in 40 μl, which was combined with an equal volume of reducing buffer and boiled for 10 min. The sample was then loaded onto an agarose gel and run overnight at 123V and 4°C, transferred to a nitrocellulose membrane, immunoblotted, and imaged using the ChemiDoc MP Imaging System to determine the isoforms (separated by size) present in the samples by comparison to in-house standards (combined material containing six apo(a) isoforms: 38, 32, 24, 19, 15, and 12 KIV-2 repeats) (29). The relative expression of each isoform was determined using the Image Lab software, which calculated relative proportions of the two isoforms based on the intensity profile of each lane. The method has an intrasample variability under 15%.

### *wIS* calculation

Most individuals express two apo(a) isoforms in plasma, and these are inversely correlated with Lp(a) plasma levels, with smaller isoforms generally dominating (13). To ascertain the contribution of isoforms to plasma Lp(a) concentration, we estimated a *wIS*. Each expressed isoform can potentially have a different FCR (equivalently, as used by some investigators, fractional synthetic rate), say, k_1_ and k_2_ for the two isoforms. As apo(a) is a slowly turning over protein and we use a primed constant infusion protocol in our studies, the apo(a) enrichment, when expressed as a fraction of the precursor plateau, goes up nearly linear during the 15 h infusion period and the rising slope, as a fraction of the plateau enrichment, equals the FCR or fractional synthetic rate. If E_1_, the enrichment of isoform 1, goes up with slope k_1_, and E_2_ goes up with slope k_2_, it can be seen that the overall enrichment E, which equals m_1_E_1_ + m_2_E_2_, goes up with slope m_1_k_1_ + m_2_k_2_, where m_1_ and m_2_ are the relative masses (i.e., mass fractions, m_1_ + m_2_ = 1) of the two isoforms, with the total mass denoted by M.

If there is a linear relationship (with intercept “a” and slope “b”) between isoform-specific FCR, termed k, and the corresponding isoform size, termed S, it means k = a + bS. Applying it to the two isoforms 1 and 2 above, the relationships become k_1_ = a + bS_1_ and k_2_ = a + bS_2_. It follows, then, that the combined apo(a) FCR, which is m_1_k_1_+m_2_k_2_, equals m_1_(a + b S_1_) + m_2_(a + bS_2_), which simplifies to a + b(m_1_S_1_ + m_2_S_2_). We define m_1_S_1_ + m_2_S_2_ as the *wIS*. Substituting, we see that the combined apo(a) FCR, termed k_c_, follows the relationship k_c_ = a + b *wIS*. Thus, when we estimate a single apo(a) FCR, it bears the same linear relationship with *wIS* as the isoform-specific FCR would bear with the corresponding isoform size. Further, if we look at the total PR, which is the sum of the two isoform-specific PR_1_ and PR_2_, where PR_1_ = m_1_Mk_1_ and PR_2_ = m_2_Mk_2_, it follows that PR = m_1_Mk_1_ + m_2_Mk_2_ = M(m_1_k_1_ + m_2_k_2_) = Mk_c_. That is, the total PR equals the total mass multiplied by the FCR we estimate from the total enrichment data. When we calculate a single apo(a) PR, it bears the same relationship with *wIS* as the isoform-specific PR would bear with the corresponding isoform size.

Example: Say the two isoform masses are M_1_ and M_2_, so total Lp(a) mass is M = M_1_ + M_2_. If the two isoform sizes are 20 and 30, with relative expression of 70% and 30%, respectively, the *wIS* is 0.7∗20+0.3∗30 = 23. If now, the two isoforms are cleared with rate constants k_1_ = 0.4 and k_2_ = 0.2, then, by the formulas above, PR_1_ = 0.7∗M∗0.4 = 0.28 M; PR_2_ = 0.3∗M∗0.2 = 0.06 M; k_c_ = 0.7∗0.4 + 0.3∗0.2 = 0.34. We see that total PR = PR_1_ + PR_2_ = 0.34 M = k_c_M.

### Apo(a) modeling

The apo(a) enrichment data were modeled as previously described (24, 25, 26). Apo(a) FCR was calculated by fitting the leucine enrichment data in an apo(a)-specific peptide using a single-pool model, with the precursor enrichment set as the VLDL apoB100 D3-leucine enrichment plateau in the same study. The plateau is typically reached during the first 15-h sampling period and estimated using our model for VLDL apoB100 metabolism (30, 31). The apo(a) PR in nmol/kg/day was calculated as the product of apo(a) FCR (in pools/day) and the apo(a) concentration (nmol/L) multiplied by the plasma volume (estimated as 0.045 L/kg).

### Statistical analysis

All data were analyzed using standard R software functions [summary, lm, estimable, ggplot, etc.] invoked by our cufunctions package (32). Variables found to be normally distributed are summarized by mean and SD, while Lp(a) levels, along with TGs, are summarized by median and interquartile range. Pearson correlation coefficients are reported. The relationship of Lp(a) levels with *wIS* in the three SRRE groups was studied by analysis of covariates (ANCOVA).

## RESULTS

The subject demographic data as well as plasma lipid and apoB100 levels are shown in Table 1. We analyzed data from 32 subjects with a mean age of 46.8 years. Seventeen subjects were female, and by SRRE, there were 17 Black, 9 Hispanic, and 6 White subjects. The mean BMI was 28.9 ± 4.3 kg/m^2^. Lipid and apoB100 levels were within normal ranges.

**Table 1:** Population demographics, lipid, and ApoB100 levels

Characteristic	Study Sample	
Age, y	46.8 ± 12.4	
Range for age	26–68	
BMI, kg/m^2^	28.9 ± 4.3	
Race (n)	Female	Male
White	2	4
Black	9	8
Hispanic	6	3
Total cholesterol, mg/dL	173 ± 38.8	
Triglycerides, mg/dL	109 (52.5, 143)	
LDL-C, mg/dL	103 ± 28.9	
HDL-C, mg/dL	51.8 ± 17.2	
Plasma ApoB100, mg/dL	81.2 ± 22.1	

Legend: ± represents Mean and Standard Deviation; ( ) represents Median and Interquartile Range.

### Lp(a) levels and Apo(a) kinetics

The study population had median Lp(a) levels of 54.6 nmol/L (interquartile range 36.8–119.0) (Table 2). Plasma Lp(a) levels did not differ between males and females (data not shown). Participants had a mean *wIS* of 22.8 ± 4. As expected from published data, Black subjects had a higher median Lp(a) concentration. In our cohort, 9 of the 32 individuals (28%) had only one detectable isoform. Mean ± SD FCR and PR of apo(a) were 0.18 ± 0.08 pools/day and 0.57 ± 0.40 nmol/kg/day, respectively (Table 2). Individual data for the full cohort are provided in supplemental Table S1.

**Table 2:** Lp(a) Plasma levels, weighted isoform size, and kinetic parameters of Apo(a)

Characteristic	Study Sample
Lp(a) (nmol/L)	54.6 (36.8, 119.0)
Black	61.2 (43.7, 127.6)
Hispanic	42.0 (21.5, 116.4)
White	49.8 (27.1, 62.3)
Weighted isoform size	22.8 ± 4.0
Apo(a) FCR (pools/day)	0.18 ± 0.1
Apo(a) PR (nmol/kg/day)	0.57 ± 0.4

PR, production rate; FCR, fractional clearance rate

Legend: ± represents Mean and Standard Deviation; ( ) represents Median and Interquartile Range.

**Fig. 1:**
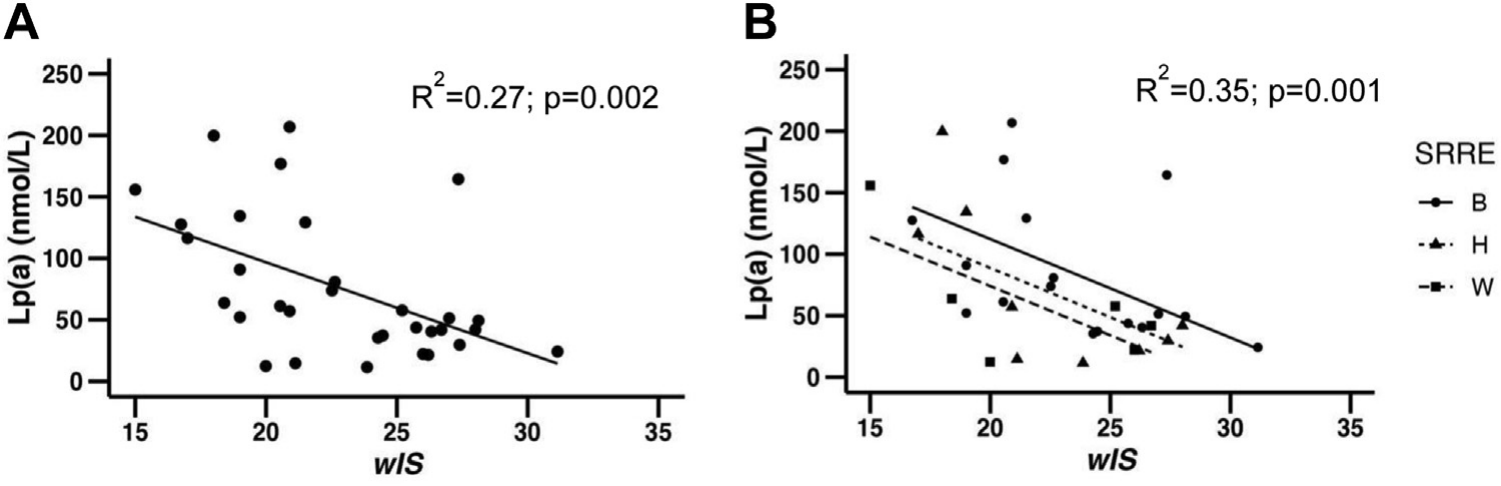
(A) Negative association of Lp(a) levels with *wIS*. B: Negative association of Lp(a) levels with *wIS*, controlling for SRRE using ANCOVA (Analysis of Covariance). Lp(a), lipoprotein(a); *wIS*, weighted isoform size; SRRE, self-reported race/ethnicity; B, Black; H, Hispanic; W, White.

As observed in larger population data sets, our subjects had an inverse relationship between Lp(a) levels and *wIS* (*R*^2^ = 0.27, *P* = 0.002) (Fig. 1A). Lp(a) levels are impacted by SRRE, hence we examined the relationship between Lp(a) levels and *wIS* for each SRRE group, Fig. 1B. Although adjustment for SRRE strengthened the overall correlation (*R*^2^ = 0.35), SRRE group differences were not statistically significant [Black-Hispanic (*P* = 0.25); Black-White (*P* = 0.11); Hispanic-White (*P* = 0.57)]. The lack of significance may be due to the small number of subjects in each SRRE group. The relationship between individual apo(a) isoforms and the Lp(a) levels associated with each isoform in the combined cohort of all subjects (Black, Hispanic, and White) was also statistically significant (*P* < 0.0001) (supplemental Fig. S1). Isoform size is a determinant of Lp(a) concentration, and it is known that SRRE plays a role in determining Lp(a) levels at any isoform size, thus we included SRRE in all our data analyses examining the relationships of *wIS* with the kinetics of apo(a).

**Fig. 2:**
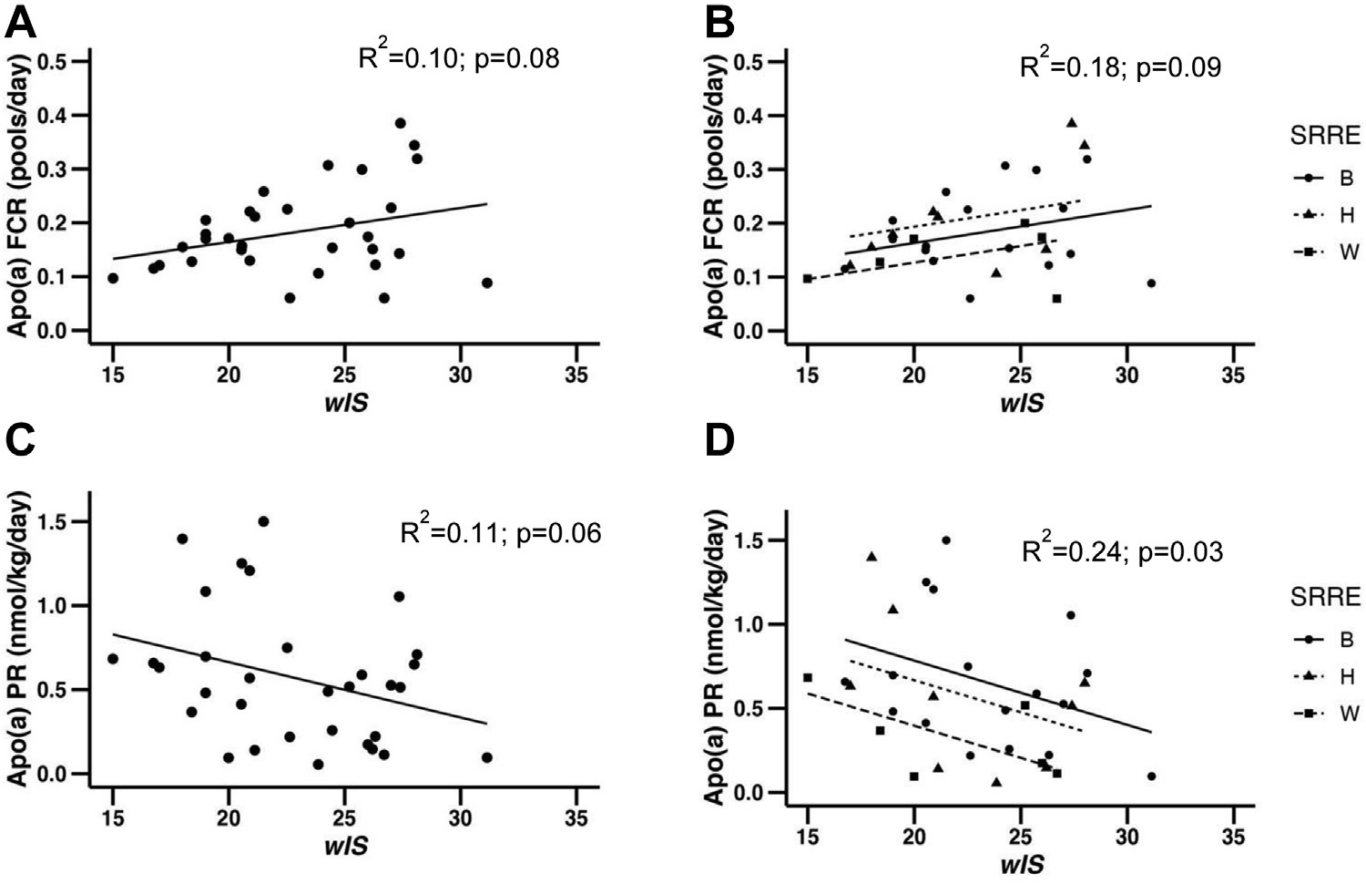
Relationship between apo(a) FCR with *wIS* before (A) and after controlling for SREE (B) using ANCOVA (Analysis of Covariance). Relationship of apo(a) PR with *wIS* before (C) and after controlling for SREE (D) using ANCOVA (Analysis of Covariance). FCR, fractional catabolic rate; PR, production rate; *wIS*, weighted isoform size; SRRE, self-reported race/ethnicity; B, Black; H, Hispanic; W, White.

The relationship between Lp(a) levels and apo(a) FCR (*R*^2^ = 0.07, *P* = 0.16) was not statistically significant (supplemental Fig. S2). When we examined the relationships of FCR with *wIS*, we found a positive trend (*P* = 0.08) (Fig. 2A). Controlling for SRRE did not affect the relationship of *wIS* with FCR (Fig. 2B).

Additionally, *wIS* showed a negative trend with apo(a) PR (*P* = 0.06) (Fig. 2C) and this relationship became statistically significant when controlling for SRRE (*P* = 0.03) (Fig. 2D).

The results above comprised all 32 subjects, including 9 individuals who expressed only a single isoform (supplemental Table S1). In this subgroup with single isoforms, we found that *wIS* and FCR were positively correlated (*R*^2^ = 0.61, *P* = 0.01) but no correlations were found between the single isoforms and PR (*R*^2^ = 0.20, *P* = 0.23) (supplemental Fig. S3*A*, *B*).

## DISCUSSION

High plasma Lp(a) levels are associated with an increased risk for ASCVD (3, 23). The pathways regulating Lp(a) levels are not well understood, and this has been recently reviewed (33, 34, 35). Similar to other cohorts (13, 14, 36, 37), the current study finds an inverse association between plasma Lp(a) levels and apo(a) allele size, with smaller isoforms associated with higher Lp(a) levels (supplemental Fig. S1). Some studies have also found smaller apo(a) isoforms to be associated with coronary artery disease (7, 38, 39, 40, 41, 42), although only two of these studies demonstrated that association to be independent of Lp(a) concentration.

### Relationship of plasma Lp(a) concentrations with PR and FCR

Previous reports support a role of production and/or clearance regulating Lp(a) plasma levels; these have been reviewed (33, 34, 35). Studies by Krempler *et al.* and Rader *et al.* found that PR, but not FCR, correlated with Lp(a) levels (17, 43). Our study agrees with these findings, as we found no significant relationship between Lp(a) levels and FCR (supplemental Fig. S2). Similarly, in a large study of the effects of a PCSK9 inhibitor on apoB metabolism in individuals without concomitant statin therapy, a treatment-associated decrease in the plasma pool size of Lp(a)-apo(a) was linked with a decrease in the PR of Lp(a)-apo(a), with no effect on FCR in the subjects not on statins. However, in the group taking statins, treatment with the PCSK9 inhibitor resulted in an increase in the FCR of Lp(a)-apo(a) with no treatment-effect on the PR of Lp(a)-apo(a) (44). These studies and other reports (21) support a role of both FCR and PR in the regulation of plasma Lp(a) levels.

### Relationship of apo(a) isoforms with PR and FCR

As previously stated, an individual’s plasma Lp(a) level, with few exceptions, is highly regulated by the number of KIV-2 repeats present in their apo(a) isoforms, which are determined by the *LPA* gene. Early studies in cultured liver cells, using both steady state labeling and pulse chase analyses, showed that the endoplasmic reticulum residence time of secreted apo(a) isoforms is determined by their size, and that this accounted for the inverse relationship between isoform size and level of secretion. The authors concluded that apo(a) posttranslational stability is a major determinant of the levels of plasma Lp(a) in baboons (45). Additional cell work provided support for the important role of the number of KIV-2 repeats in the rate of assembly and secretion of apo(a) (46, 47, 48). Human studies using externally labeled Lp(a) demonstrated the importance of PR in determining plasma levels of Lp(a) in subjects with varying Lp(a) levels and either similar apo(a) isoforms (17) or varying apo(a) sizes (18). The latter study showed an inverse correlation between apo(a) size and PR of apo(a). In both of these studies, the FCRs of Lp(a) were not related to the concentration of plasma Lp(a). On the other hand, Jenner *et al.* reported that isoform size, determined by gel electrophoretic separation, affected both the PR and FCR of apo(a) in studies using endogenous labeling of Lp(a) with stable isotopes (21). Subjects with smaller isoforms had higher PRs of apo(a), similar to the findings of Rader *et al.*, but they also had lower apo(a) FCRs, the latter similar to our current findings. These previous studies did not determine and take into account participant SRRE.

Advances in mass spectrometry and methods to isolate Lp(a) have enhanced our ability to interrogate the mechanisms that regulate Lp(a) (33). In this current study, we used *wIS* (see Methods), which captures the contribution of each isoform to the Lp(a) level in the circulation. The use of isoform expression to calculate isoform specific Lp(a) plasma levels has been applied in earlier studies (38). Calculation of the *wIS* suffers from some limitations listed below, yet it allowed us to assess the effects of a weighted mean of two expressed isoforms on FCR and PR of apo(a). We found that *wIS* had only modest correlations with both PR and FCR. Our results are consistent with previous reports that found strong relationships between allele size and PR but also identified trends with FCR (17, 18, 19, 21, 49, 50). As seen in Fig. 2A, one individual in our cohort had a very large *wIS* and excluding this individual from the analysis improved the relationship between *wIS* and FCR (*P* < 0.02). Relevant to our current findings, a sub-analysis by Chan *et al.* (19) of the baseline results obtained from a study of the effects of evolocumab on the kinetic of Lp(a) metabolism, found that levels of Lp(a) were negatively associated with apo(a) size and FCR and positively associated with PR. Moreover, in subjects with small isoforms (≤22 KIV-2), they found strong correlations between apo(a) concentration and increased apo(a) PR but not with FCR. In subjects with large isoforms (>22 KIV-2), on the other hand, Lp(a) levels were correlated with both kinetic parameters (19). The authors found similar associations in the subjects treated with either statin alone, evolocumab alone, or the combination of the two treatments. They demonstrated that Lp(a) lowering with a PCSK9 inhibitor, evolocumab, lowered plasma Lp(a) levels by decreasing apo(a) PR and increasing apo(a) FCR. We found similar results when administering the PCSK9 inhibitor, alirocumab (25). Importantly, different nontargeted Lp(a)-lowering treatments decrease Lp(a) by different effects on FCR and PR. Niacin lowered plasma Lp(a) levels in association with decrease in both PR and FCR (51). Mipomersen, an apoB antisense oligonucleotide (ASO), reduced Lp(a) by increasing FCR, although PR was reduced as well in some individuals (24). Anacetrapib, a CETP inhibitor, decreased Lp(a) by decreasing PR (26). The results from those studies support a complex regulatory mechanism of Lp(a) levels. This may be due to the additional proteins and lipids found on and within Lp(a) particles (52). Lastly, the exact location where the covalent linkage of apo(a) to apoB100 assembly occurs (intrahepatic or at the surface of the liver), as well as the site and molecular mechanism of Lp(a) clearance from plasma are not completely defined (33, 34, 35). Recent studies using cell models with a single isoform (17 KIV-2 repeats), found that, in addition to a covalent disulfide bond between apo(a) and apoB100, there are also noncovalent interactions between these two proteins (53). The latter observations, if true in vivo, could affect measurements of FCR. Additionally, free apo(a) fragments have been found in plasma and urine but their concentrations are very low and their physiological role, if any, were poorly understood (54, 55).

Due to clear racial differences in the relationship of isoform size and plasma Lp(a) levels (56, 57), it is important to control for these when analyzing such data. Since our study population was composed of a diverse cohort, we controlled for any effects of SRRE on the analyzed study outcomes. When we adjusted for SRRE, the relationship between *wIS* and PR was statistically significant.

Lp(a) lowering with nontargeted and targeted treatments decreases both isoforms. Of interest, the relative expression of apo(a) isoforms does not change after Lp(a) levels are lowered using ASO apo(a) treatment (58). The latter result suggests that apo(a) ASO treatment does not preferentially affect one isoform size over the other. Similarly, in data from our lab, we have not observed treatment effects on *wIS* after various therapies that lower apoB100 and apo(a) (supplemental Table S2). A recent study using PCSK9 inhibitors showed a positive correlation between apo(a) size and reductions in Lp(a) levels for both small and large isoforms of apo(a) (59). Recent studies using a targeted siRNA therapy showed significant Lp(a) lowering but no isoform size data have been presented (60, 61) .

Lastly, there are studies examining the roles of single nucleotide polymorphisms (SNP) present in the *LPA* gene within the KIV2 region that are linked to high and low Lp(a) levels. The allele frequencies of these SNPs have been found to differ across SRRE groups (22, 62, 63). These differences in SNP presentation could explain why Lp(a) levels differ for similar isoform sizes in different SRRE groups. The effect of these SNP on the clearance and production of Lp(a) has not been studied.

Study Limitations: Our results indicate that apo(a) isoforms have a significant yet modest contribution to the mechanisms regulating apo(a) FCR and PR. Although we did include subjects with different SRRE in this study, our study population was small, with 17 of our 32 subjects identifying as Black, leaving very few subjects in the other groups. Mechanistic studies are costly and labor extensive, hence it will be difficult to perform studies in large populations with adequate sample size for different SRRE groups. Our results, however, highlight the need to recruit diverse cohorts when designing these studies. Berglund *et al.* examined the role of isoforms in larger diverse cohorts (56) showing associations similar to those found in our cohort, with Blacks having higher Lp(a) level for the same apo(a) isoform size than Whites, even though the difference did not reach statistical significance in our study due to the limited subject number. However, the study by Berglund *et al.* did not examine metabolic pathways (56).

There were methodologic limitations: In the current study, we did not isolate individual apo(a) isoforms and calculate their unique FCR and PR; instead, we used the relative expression data from gel electrophoresis to estimate their contributions to the *wIS*. We examined the kinetics of apo(a) isolated from LDL or LDL+HDL fractions. However, the apo(a) PR and FCR from LDL-only or from LDL plus HDL fractions were not statistically different (supplemental Fig. S4) and the data were, therefore, combined for all analyses. Apo(a) measurements were performed on plasma samples by a validated ELISA (28) and not on the mass spectrometry used to obtain enrichments. Various methods have been proposed to measure apo(a) via mass spectrometry (64), however we did not have these methods available at the time of the study.

## Data availability

All the data generated during and/or analyzed during the current study are available from the corresponding author and are included in this published article and its supplementary information file.

## Supplemental data

This article contains supplemental data (25, 65, 66).

## Conflict of interest

The authors declare that they have no conflicts of interest with the contents of this article

## References

[1] Berg K. (1963). A new serum type system in man-the LP system. Acta Pathol. Microbiol. Scandanavia.

[2] Jawi M.M., Frohlich J., Chan S.Y. (2020). Lipoprotein(a) the insurgent: a New insight into the structure, function, metabolism, pathogenicity, and medications affecting lipoprotein(a) molecule. J. Lipids.

[3] Reyes-Soffer G., Ginsberg H.N., Berglund L., Duell P.B., Heffron S.P., Kamstrup P.R. (2022). American Heart Association Council on Arteriosclerosis T, Vascular B, Council on Cardiovascular R, Intervention and Council on Peripheral Vascular D. Lipoprotein(a): a genetically determined, Causal, and prevalent risk factor for Atherosclerotic Cardiovascular Disease: a scientific statement from the American Heart Association. Arterioscler Thromb. Vasc. Biol..

[4] Nordestgaard B.G., Langsted A. (2016). Lipoprotein (a) as a cause of cardiovascular disease: insights from epidemiology, genetics, and biology. J. Lipid Res..

[5] Kettunen J., Demirkan A., Wurtz P., Draisma H.H., Haller T., Rawal R. (2016). Genome-wide study for circulating metabolites identifies 62 loci and reveals novel systemic effects of LPA. Nat. Commun..

[6] Mack S., Coassin S., Rueedi R., Yousri N.A., Seppala I., Gieger C. (2017). A genome-wide association meta-analysis on lipoprotein (a) concentrations adjusted for apolipoprotein (a) isoforms. J. Lipid Res..

[7] Saleheen D., Haycock P.C., Zhao W., Rasheed A., Taleb A., Imran A. (2017). Apolipoprotein(a) isoform size, lipoprotein(a) concentration, and coronary artery disease: a mendelian randomisation analysis. Lancet Diabetes Endocrinol..

[8] Burgess S., Ference B.A., Staley J.R., Freitag D.F., Mason A.M., Nielsen S.F. (2018). European prospective investigation into Cancer Nutrition-Cardiovascular Disease Consortium. Association of LPA variants with risk of Coronary Disease and the implications for lipoprotein(a)-lowering therapies: a mendelian randomization analysis. JAMA Cardiol..

[9] Lackner C., Boerwinkle E., Leffert C.C., Rahmig T., Hobbs H.H. (1991). Molecular basis of apolipoprotein(a) isoform size heterogeneity as revealed by pulsed-field get electrophoresis. J. Clin. Invest..

[10] Scanu A.M. (1988). Lipoprotein(a): a genetically determined lipoprotein containing a glycoprotein of the plasminogen family. Semin. Thromb. Hemost..

[11] Kostner K.M., Kostner G.M. (2017). Lipoprotein (a): a historical appraisal. J. Lipid Res..

[12] Utermann G. (1999). Genetic architecture and evolution of the lipoprotein(a) trait. Curr. Opin. Lipidol..

[13] Kraft H.G., Sandholzer C., Menzel H.J., Utermann G. (1992). Apolipoprotein (a) alleles determine lipoprotein (a) particle density and concentration in plasma. Arterioscler. Thromb..

[14] Sandholzer C., Saha N., Kark J.D., Rees A., Jaross W., Dieplinger H. (1992). Apo(a) isoforms predict risk for coronary heart disease. A study in six populations. Arterioscler Thromb..

[15] Schmidt K.N.A., Kronenberg F., Utermann G. (2016). Structure, function, and genetics of lipoprotein(a). J. Lipid Res..

[16] Frischmann M.E., Kronenberg F., Trenkwalder E., Schaefer J.R., Schweer H., Dieplinger B. (2007). In vivo turnover study demonstrates diminished clearance of lipoprotein(a) in hemodialysis patients. Kidney Int..

[17] Rader D.J., Cain W., Zech L.A., Usher D., Brewer H.B.J. (1993). Variation in lipoprotein (a) concentrations among individuals with the same apolipoprotein (a) isoform is determined by the rate of lipoprotein (a) production. J. Clin. Invest..

[18] Rader D.J., Cain W., Ikewaki K., Talley G., Zech L.A., Usher D. (1994). The inverse association of plasma lipoprotein(a) concentrations with apolipoprotein(a) isoform size is not due to differences in Lp(a) catabolism but to differences in production rate. J. Clin. Invest..

[19] Chan D.C., Watts G.F., Coll B., Wasserman S.M., Marcovina S.M., Barrett P.H.R. (2019). Lipoprotein(a) particle production as a determinant of plasma lipoprotein(a) concentration across varying apolipoprotein(a) isoform sizes and background cholesterol-lowering therapy. J. Am. Heart Assoc..

[20] Ma L., Chan D.C., Ooi E.M.M., Marcovina S.M., Barrett P.H.R., Watts G.F. (2019). Apolipoprotein(a) kinetics in statin-treated patients with elevated plasma lipoprotein(a) concentration. J. Clin. Endocrinol. Metab..

[21] Jenner J.L., Seman L.J., Millar J.S., Lamon-Fava S., Welty F.K., Dolnikowski G.G. (2005). The metabolism of apolipoproteins (a) and B-100 within plasma lipoprotein (a) in human beings. Metabolism.

[22] Enkhmaa B., Anuurad E., Berglund L. (2016). Lipoprotein (a): impact by ethnicity and environmental and medical conditions. J. Lipid Res..

[23] Patel A.P., Wang M., Pirruccello J.P., Ellinor P.T., Ng K., Kathiresan S. (2021). Lp(a) (Lipoprotein[a]) concentrations and incident Atherosclerotic Cardiovascular Disease: new insights from a large national biobank. Arterioscler. Thromb. Vasc. Biol..

[24] Nandakumar R., Matveyenko A., Thomas T., Pavlyha M., Ngai C., Holleran S. (2018). Effects of mipomersen, an apolipoprotein B100 antisense, on lipoprotein (a) metabolism in healthy subjects. J. Lipid Res..

[25] Reyes-Soffer G., Pavlyha M., Ngai C., Thomas T., Holleran S., Ramakrishnan R. (2017). Effects of PCSK9 inhibition with alirocumab on lipoprotein metabolism in healthy humans. Circulation.

[26] Thomas T., Zhou H., Karmally W., Ramakrishnan R., Holleran S., Liu Y. (2017). CETP (Cholesteryl Ester Transfer Protein) inhibition with anacetrapib decreases production of lipoprotein(a) in mildly hypercholesterolemic subjects. Arterioscler. Thromb. Vasc. Biol..

[27] Zhou H., Castro-Perez J., Lassman M.E., Thomas T., Li W., McLaughlin T. (2013). Measurement of apo(a) kinetics in human subjects using a microfluidic device with tandem mass spectrometry. Rapid Commun. Mass Spectrom..

[28] Marcovina S.M., Albers J.J., Scanu A.M., Kennedy H., Giaculli F., Berg K. (2000). Use of a reference material proposed by the International Federation of Clinical Chemistry and Laboratory Medicine to evaluate analytical methods for the determination of plasma lipoprotein(a). Clin. Chem..

[29] Marcovina S.M., Hobbs H.H., Albers J.J. (1996). Relation between number of apolipoprotein(a) kringle 4 repeats and mobility of isoforms in agarose gel: basis for a standardized isoform nomenclature. Clin. Chem..

[30] Ramakrishnan R., Ramakrishnan J.D. (2008). Using mass measurements in tracer studies--a systematic approach to efficient modeling. Metabolism.

[31] Ramakrishnan R. (2006). Studying apolipoprotein turnover with stable isotope tracers: correct analysis is by modeling enrichments. J. Lipid Res..

[32] Holleran S., Ramakrishnan R. (2021). http://biomath.net/cufunctions.html.

[33] Reyes-Soffer G., Ginsberg H.N., Ramakrishnan R. (2017). The metabolism of lipoprotein (a): an ever-evolving story. J. Lipid Res..

[34] Boffa M.B., Koschinsky M.L. (2022). Understanding the ins and outs of lipoprotein (a) metabolism. Curr. Opin. Lipidol..

[35] Chemello K., Chan D.C., Lambert G., Watts G.F. (2022). Recent advances in demystifying the metabolism of lipoprotein(a). Atherosclerosis.

[36] Gavish D., Azrolan N., Breslow J.L. (1989). Plasma Ip(a) concentration is inversely correlated with the ratio of Kringle IV/Kringle V encoding domains in the apo(a) gene. J. Clin. Invest..

[37] Boerwinkle E., Leffert C.C., Lin J., Lackner C., Chiesa G., Hobbs H.H. (1992). Apolipoprotein(a) gene accounts for greater than 90% of the variation in plasma lipoprotein(a) concentrations. J. Clin. Invest..

[38] Paultre F., Pearson T.A., Weil H.F., Tuck C.H., Myerson M., Rubin J. (2000). High levels of Lp(a) with a small apo(a) isoform are associated with coronary artery disease in African American and white men. Arterioscler. Thromb. Vasc. Biol..

[39] Zeljkovic A., Bogavac-Stanojevic N., Jelic-Ivanovic Z., Spasojevic-Kalimanovska V., Vekic J., Spasic S. (2009). Combined effects of small apolipoprotein (a) isoforms and small, dense LDL on coronary artery disease risk. Arch. Med. Res..

[40] Kamstrup P.R., Tybjaerg-Hansen A., Steffensen R., Nordestgaard B.G. (2009). Genetically elevated lipoprotein(a) and increased risk of myocardial infarction. JAMA.

[41] Ooi E.M., Ellis K.L., Barrett P.H.R., Watts G.F., Hung J., Beilby J.P. (2018). Lipoprotein(a) and apolipoprotein(a) isoform size: associations with angiographic extent and severity of coronary artery disease, and carotid artery plaque. Atherosclerosis.

[42] Gudbjartsson D.F., Thorgeirsson G., Sulem P., Helgadottir A., Gylfason A., Saemundsdottir J. (2019). Lipoprotein(a) concentration and risks of Cardiovascular Disease and Diabetes. J. Am. Coll. Cardiol..

[43] Krempler F., Kostner G.M., Bolzano K., Sandhofer F. (1980). Turnover of lipoprotein (a) in man. J. Clin. Invest..

[44] Watts G.F., Chan D.C., Somaratne R., Wasserman S.M., Scott R., Marcovina S.M. (2018). Controlled study of the effect of proprotein convertase subtilisin-kexin type 9 inhibition with evolocumab on lipoprotein(a) particle kinetics. Eur. Heart J..

[45] White A.L., Rainwater D.L., Hixson J.E., Estlack L.E., Lanford R.E. (1994). Intracellular processing of apo(a) in primary baboon hepatocytes. Chem. Phys. Lipids.

[46] Brunner C., Lobentanz E.M., Petho-Schramm A., Ernst A., Kang C., Dieplinger H. (1996). The number of identical kringle IV repeats in apolipoprotein(a) affects its processing and secretion by HepG2 cells. J. Biol. Chem..

[47] Bonen D.K., Hausman A.M., Hadjiagapiou C., Skarosi S.F., Davidson N.O. (1997). Expression of a recombinant apolipoprotein(a) in HepG2 cells. Evidence for intracellular assembly of lipoprotein(a). J. Biol. Chem..

[48] Lobentanz E.M., Krasznai K., Gruber A., Brunner C., Muller H.J., Sattler J. (1998). Intracellular metabolism of human apolipoprotein(a) in stably transfected Hep G2 cells. Biochemistry.

[49] Watts G.F., Chan D.C., Pang J., Ma L., Ying Q., Aggarwal S. (2020). PCSK9 Inhibition with alirocumab increases the catabolism of lipoprotein(a) particles in statin-treated patients with elevated lipoprotein(a). Metabolism.

[50] Ying Q., Chan D.C., Pang J., Marcovina S.M., Barrett P.H.R., Watts G.F. (2022). PCSK9 inhibition with alirocumab decreases plasma lipoprotein(a) concentration by a dual mechanism of action in statin-treated patients with very high apolipoprotein(a) concentration. J. Intern. Med..

[51] Croyal M., Ouguerram K., Passard M., Ferchaud-Roucher V., Chetiveaux M., Billon-Crossouard S. (2015). Effects of extended-release nicotinic acid on apolipoprotein (a) kinetics in hypertriglyceridemic patients. Arterioscler. Thromb. Vasc. Biol..

[52] von Zychlinski A., Kleffmann T., Williams M.J., McCormick S.P. (2011). Proteomics of Lipoprotein(a) identifies a protein complement associated with response to wounding. J. Proteomics.

[53] Youssef A., Clark J.R., Marcovina S.M., Boffa M.B., Koschinsky M.L. (2022). Apo(a) and ApoB interact noncovalently within hepatocytes: implications for regulation of Lp(a) levels by modulation of ApoB secretion. Arterioscler. Thromb. Vasc. Biol..

[54] Mooser V., Seabra M.C., Abedin M., Landschulz K.T., Marcovina S., Hobbs H.H. (1996). Apolipoprotein(a) kringle 4-containing fragments in human urine. Relationship to plasma levels of lipoprotein(a). J. Clin. Invest..

[55] Doucet C., Mooser V., Gonbert S., Raymond F., Chapman J., Jacobs C. (2000). Lipoprotein(a) in the nephrotic syndrome: molecular analysis of lipoprotein(a) and apolipoprotein(a) fragments in plasma and urine. J. Am. Soc. Nephrol..

[56] Berglund L., Kim K., Zhang W., Prakash N., Truax K., Anuurad E. (2021). Lp(a)-Associated oxidized phospholipids in healthy black and white participants in relation to apo(a) size, age, and family structure. J. Am. Heart Assoc..

[57] Rubin J., Paultre F., Tuck C.H., Holleran S., Reed R.G., Pearson T.A. (2002). Apolipoprotein [a] genotype influences isoform dominance pattern differently in African Americans and Caucasians. J. Lipid Res..

[58] Karwatowska-Prokopczuk E., Clouet-Foraison N., Xia S., Viney N.J., Witztum J.L., Marcovina S.M. (2021). Prevalence and influence of LPA gene variants and isoform size on the Lp(a)-lowering effect of pelacarsen. Atherosclerosis.

[59] Blanchard V., Chemello K., Hollstein T., Hong-Fong C.C., Schumann F., Grenkowitz T. (2022). The size of apolipoprotein (a) is an independent determinant of the reduction in lipoprotein (a) induced by PCSK9 inhibitors. Cardiovasc. Res..

[60] O'Donoghue M.L., Rosenson R.S., Gencer B., Lopez J.A.G., Lepor N.E., Baum S.J. (2022). Small interfering RNA to reduce lipoprotein(a) in Cardiovascular Disease. N. Engl. J. Med..

[61] Nissen S.E., Wolski K., Balog C., Swerdlow D.I., Scrimgeour A.C., Rambaran C. (2022). Single ascending dose study of a short interfering RNA targeting lipoprotein(a) production in individuals with elevated plasma lipoprotein(a) levels. JAMA.

[62] Dumitrescu L., Glenn K., Brown-Gentry K., Shephard C., Wong M., Rieder M.J. (2011). Variation in LPA is associated with Lp(a) levels in three populations from the Third National Health and Nutrition Examination Survey. PLoS One.

[63] Zekavat S.M., Ruotsalainen S., Handsaker R.E., Alver M., Bloom J., Poterba T. (2018). Deep coverage whole genome sequences and plasma lipoprotein(a) in individuals of European and African ancestries. Nat. Commun..

[64] Lassman M.E., McLaughlin T.M., Zhou H., Pan Y., Marcovina S.M., Laterza O. (2014). Simultaneous quantitation and size characterization of apolipoprotein(a) by ultra-performance liquid chromatography/mass spectrometry. Rapid Commun. Mass Spectrom..

[65] Reyes-Soffer G., Millar J., Ngai C., Jumes P., Coromilas E., Asztalos B. (2016). Cholesteryl ester transfer protein inhibition with anacetrapib decreases fractional clearance rates of high-density lipoprotein apolipoprotein A-I and plasma cholesteryl ester transfer protein. Artheroscler Thromb Vasc Biol.

[66] Reyes-Soffer G., Moon B., Hernandez-Ono A., Dionizovick-Dimanovski M., Jimenez J., Obunike J. (2016). Complex effects of inhibiting hepatic apolipoprotein B100 synthesis in humans. Sci Transl Med.

