# Supplementary material for: Relationship of apolipoprotein(a) isoform size with clearance and production of lipoprotein(a) in a diverse cohort
Source: J Lipid Res. 2023 Jan 24;64(3):100336. doi: 10.1016/j.jlr.2023.100336 (PMC10006688; doi:10.1016/j.jlr.2023.100336)
Supplement: Supplemental data [file mmc1.docx]

**Supplemental Tables and Figures**

**Supplemental Table 1.** Individual Subject Data

| Subject # | *wIS* | Smaller Apo(a) isoform | % smaller | Larger Apo(a) isoform | % larger | Lp(a) (nmol/L) | Apo(a) FCR | Apo(a)  PR | SRRE |
| --- | --- | --- | --- | --- | --- | --- | --- | --- | --- |
| 1* | 15 | 15 | 100 |  | 0 | 155.8 | 0.1 | 0.68 | W |
| 2 | 16.8 | 15 | 75 | 22 | 25 | 127.6 | 0.12 | 0.66 | B |
| 3* | 17 | 17 | 100 |  | 0 | 116.4 | 0.12 | 0.63 | H |
| 4* | 18 | 18 | 100 |  | 0 | 199.7 | 0.16 | 1.4 | H |
| 5 | 18.4 | 18 | 90 | 22 | 10 | 63.8 | 0.13 | 0.37 | W |
| 6* | 19 | 19 | 100 |  | 0 | 134.4 | 0.18 | 1.08 | H |
| 7* | 19 | 19 | 100 |  | 0 | 52.2 | 0.21 | 0.48 | B |
| 8* | 19 | 19 | 100 |  | 0 | 90.9 | 0.17 | 0.7 | B |
| 9 | 20 | 19 | 67 | 22 | 33 | 12.4 | 0.17 | 0.1 | W |
| 10 | 20.5 | 20 | 73 | 22 | 27 | 61.2 | 0.15 | 0.41 | B |
| 11 | 20.6 | 19 | 22 | 21 | 78 | 176.9 | 0.16 | 1.25 | B |
| 12 | 20.9 | 20 | 70 | 23 | 30 | 57.1 | 0.22 | 0.57 | H |
| 13 | 20.9 | 20 | 70 | 23 | 30 | 206.8 | 0.13 | 1.21 | B |
| 14 | 21.1 | 20 | 84 | 27 | 16 | 14.7 | 0.21 | 0.14 | H |
| 15 | 21.5 | 20 | 50 | 23 | 50 | 129.2 | 0.26 | 1.5 | B |
| 16 | 22.5 | 21 | 81 | 29 | 19 | 73.9 | 0.23 | 0.75 | B |
| 17 | 22.6 | 22 | 79 | 25 | 21 | 80.8 | 0.06 | 0.22 | B |
| 18 | 23.9 | 19 | 73 | 37 | 27 | 11.5 | 0.11 | 0.06 | H |
| 19 | 24.3 | 19 | 12 | 25 | 88 | 35.4 | 0.31 | 0.49 | B |
| 20 | 24.5 | 22 | 18 | 25 | 82 | 37.3 | 0.15 | 0.26 | B |
| 21 | 25.2 | 23 | 80 | 34 | 20 | 57.7 | 0.2 | 0.52 | W |
| 22 | 25.7 | 25 | 63 | 27 | 37 | 43.7 | 0.3 | 0.59 | B |
| 23* | 26 | 26 | 100 |  | 0 | 22.2 | 0.17 | 0.17 | W |
| 24 | 26.2 | 21 | 60 | 34 | 40 | 21.5 | 0.15 | 0.15 | H |
| 25 | 26.3 | 24 | 42 | 28 | 58 | 40.5 | 0.12 | 0.22 | B |
| 26 | 26.7 | 26 | 65 | 28 | 35 | 41.9 | 0.06 | 0.11 | W |
| 27* | 27 | 27 | 100 |  | 0 | 51.3 | 0.23 | 0.53 | B |
| 28 | 27.4 | 26 | 55 | 29 | 45 | 164.4 | 0.14 | 1.05 | B |
| 29 | 27.4 | 23 | 60 | 34 | 40 | 29.7 | 0.39 | 0.51 | H |
| 30* | 28 | 28 | 100 |  | 0 | 42 | 0.34 | 0.65 | H |
| 31 | 28.1 | 27 | 44 | 29 | 56 | 49.4 | 0.32 | 0.71 | B |
| 32 | 31.1 | 30 | 62 | 33 | 38 | 24.2 | 0.09 | 0.1 | B |

Legend: *wIS* – weighted Isoform Size; FCR – Fractional Catabolic Rate (pools/day); PR – Production Rate (nmol/kg/day); SRRE – Self Reported Race Ethnicity; * - These subjects had only one isoform expressed.

**Supplemental Table 2.** Effects of Apolipoprotein B100 and LDL-C Lowering Treatments on apo(a) Weighted Isoform Size.

|  | | |  |  |
| --- | --- | --- | --- | --- |
|  |  |  |  |  |
|  | **Entire Cohort (n=32)** | **CETP Inhibitor^1^ (n=6)** | **ApoB100 Inhibitor^2^ (n=10)** | **PCSK9 Inhibitor^3^ (n=16)** |
| ***wIS*** | Pre-T: 22.8±4.0  Post-T: 22.5±4.0 | Pre-T: 24.2±4.5  Post-T: 23.6±4.3 | Pre-T: 21.5±4.4  Post-T: 21.4±4.4 | Pre-T: 23.1±3.6  Post-T: 22.8±3.7 |
| **p-value** | 0.11 | 0.38 | 0.39 | 0.3 |
|  |  |  |  |  |
| Legend: Paired t.test p-values of pre and post *wIS* for our entire cohort and by study; Pre-T: Pre-Treatment mean isoform size ± standard deviation; Post-T: Post Treatment mean isoform size ± standard deviation  *wIS*: weighted Isoform Size.  CETP: Cholesteryl ester transfer protein  PCSK9: Proprotein convertase subtilisin/kexin type 9 | | | |  |

**Supplemental Figure 1.** Relationship Between Apo(a) Isoform Size and Isoform Specific Lp(a) Levels in Blacks, Hispanics, and Whites.


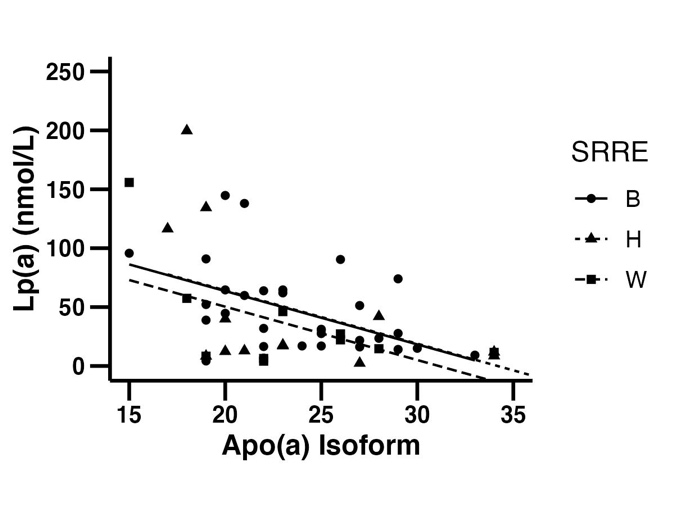


R^2^=0.27; p<0.0001

Legend: Negative relationship of individual apo(a) isoforms of all study subjects with isoform specific Lp(a) levels. Isoform size was estimated by imaging of agarose gels after plasma electrophoresis. Apo(a) Isoform concentrations were calculated by combining isoform size and relative mass from agarose gels: For example: subject with 20 and 30 Apo(a) isoforms with 50/50% expression and 300 nmol/L total Lp(a) level (measured separately by ELISA) – would result in two points (x,y) – (20,150) and (30,150). The regression lines for Blacks and Hispanics overlapped. Lp(a) – lipoprotein(a); SSRE - Self-Reported Race Ethnicity: B – Black, H – Hispanic, W – White.

**Supplemental Figure 2.** Relationship between Plasma Lp(a) levels and Apo(a) FCR


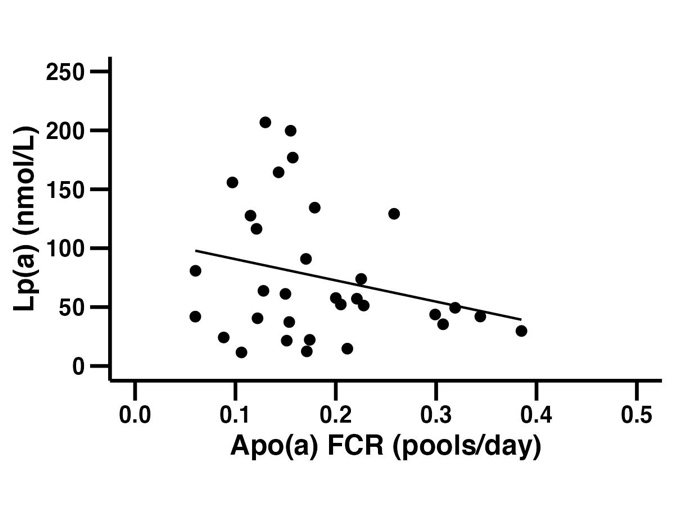


R^2^=0.07; p=0.16

Legend: Lp(a) levels were not associated with apo(a) FCR. Lp(a) – lipoprotein(a); FCR - Fractional Catabolic Rate.

**Supplemental Figure 3.** Relationship Between *wIS* and FCR (A) and PR (B) in Subjects with Single Expressed Apo(a) Isoforms


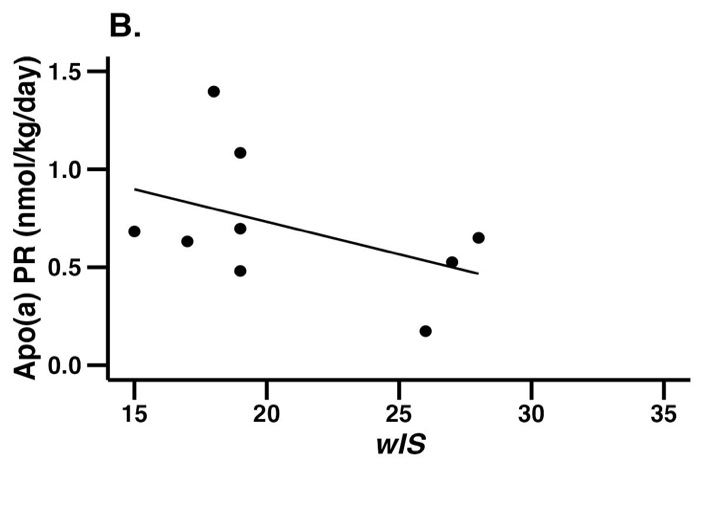


R^2^=0.20; p=0.23


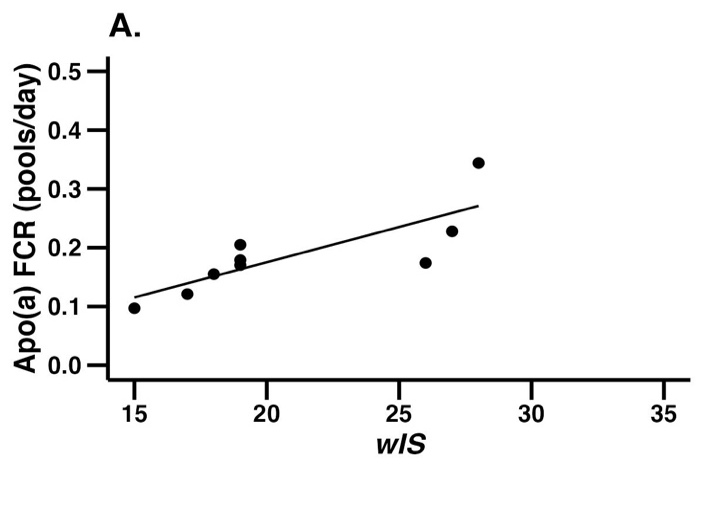


R^2^=0.65; p=0.009

Legend: In individuals expressing one single apo(a) isoform; *wIS* was positively correlated with apo(a) FCR (A) and not correlated with apo(a) PR (B). *wIS* – weighted Isoform Size; FCR - Fractional Catabolic Rate; PR – Production Rate; n = 9 subjects.

**Supplemental Figure 4.** Relationship Between *wIS* and FCR in Lipoprotein Fractions


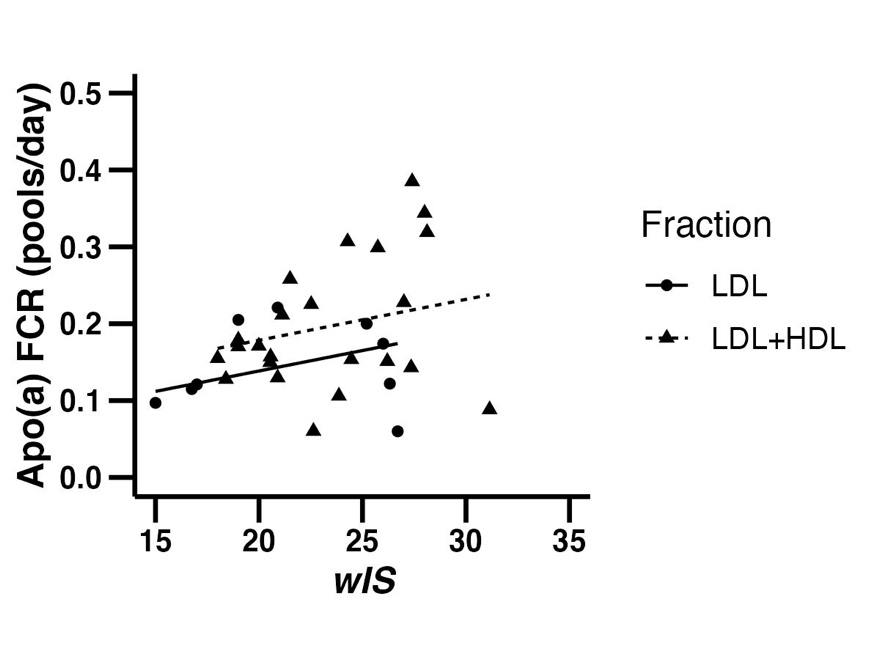


R^2^=0.15; p=0.145

Legend: Statistical analysis showed no statistically significant difference between LDL+HDL and LDL only fractions when examining at the relationship of apo(a) FCR and wIS (p=0.21).

*wIS* – weighted Isoform Size; FCR – Fractional Catabolic Rate; LDL-Low Density Lipoprotein; HDL-High Density Lipoprotein.

References:

1. Reyes-Soffer G, Millar J, Ngai C, Jumes P, Coromilas E, Asztalos B, Johnson-Levonas A, Wagner J, Donovan D, Karmally W, Ramakrishnan R, Holleran S, Thomas T, Dunbar R, deGoma E, Rafeek H, Baer A, Liu Y, Lassman M, Gutstein D, Rader D and Ginsberg H. Cholesteryl Ester Transfer Protein Inhibition With Anacetrapib Decreases Fractional Clearance Rates of High-Density Lipoprotein Apolipoprotein A-I and Plasma Cholesteryl Ester Transfer Protein. *Artheroscler Thromb Vasc Biol*. 2016;36:994-1002.

2. Reyes-Soffer G, Moon B, Hernandez-Ono A, Dionizovick-Dimanovski M, Jimenez J, Obunike J, Thomas T, Ngai C, Fontanez N, Donovan DS, Karmally W, Holleran S, Ramakrishnan R, Mittleman RS and Ginsberg HN. Complex effects of inhibiting hepatic apolipoprotein B100 synthesis in humans. *Sci Transl Med*. 2016;8:323ra12.

3. Reyes-Soffer G, Pavlyha M, Ngai C, Thomas T, Holleran S, Ramakrishnan R, Karmally W, Nandakumar R, Fontanez N, Obunike J, Marcovina SM, Lichtenstein AH, Matthan NR, Matta J, Maroccia M, Becue F, Poitiers F, Swanson B, Cowan L, Sasiela WJ, Surks HK and Ginsberg HN. Effects of PCSK9 Inhibition With Alirocumab on Lipoprotein Metabolism in Healthy Humans. *Circulation*. 2017;135:352-362.
